# Influence of Ethanol on Porous Anodic Alumina Growth in Etidronic Acid Solutions at Various Temperatures

**DOI:** 10.3390/ma15238595

**Published:** 2022-12-02

**Authors:** Małgorzata Kwiatkowska, Dariusz Siemiaszko, Małgorzata Norek

**Affiliations:** Institute of Materials Science and Engineering, Faculty of Advanced Technologies and Chemistry, Military University of Technology, 2, 00-908 Warsaw, Poland

**Keywords:** porous anodic alumina (PAA), etidronic acid, anodization, ethanol, large cell size

## Abstract

Etidronic acid, used in aluminum anodization, has a great potential for the fabrication of porous anodic alumina (PAA) with large cell sizes (>540 nm). PAAs are particularly suited to applications in optics and photonics where large-scale periodicity corresponding to visible or infrared light is needed. Additionally, such PAAs should be characterized by long-range pore ordering. However, to obtain regular pore arrangement in an etidronic electrolyte, the anodization should be performed at high electric fields using relatively high temperatures, which makes the process challenging in terms of its stability. To stabilize the process, the electrolyte can be modified with ethanol. In this work, the impact of ethanol on pore geometry and a level of pore ordering is systematically analyzed. It is shown that the additive tends to reduce pore ordering. Moreover, by changing the anodizing temperature and the amount of ethanol, it is possible to tune the porosity of the PAA template. At 20 °C, porosity drops from 14% in PAA grown in a pure water-based electrolyte to ca. 8% in PAA fabricated in the 1:*3 v*/*v* EtOH:H_2_O electrolyte. The larger PAA thickness obtained for the same charge density strongly suggests that PAA formation efficiency increases in the 1:3 *v*/*v* EtOH:H_2_O mixture.

## 1. Introduction

Porous anodic alumina (PAA) is a well-known template for the synthesis of different nanostructures [1] for a variety of applications, including photocatalysis [2,3,4], supercapacitors [5], optical circuits [6], communication devices [7], or sensing [8,9]. Regular growth of pores depends on a subtle tuning of electrochemical conditions which define pore diameter (D_p_), interpore distance (D_c_) or PAA thickness. PAA templates with highly regular pores arranged into a close-packed hexagonal (hcp) structure, and with D_c_ (cell size) that correspond to visible-IR light wavelengths, are required for applications in fields such as photonics or plasmonics [10,11]. However, the hcp structure can be obtained solely within the so-called self-organization regime, which, for a given electrolyte, holds within narrow anodization windows. Usually, these windows are limited to a narrow voltage range dependent on the acid species. It was shown that the anodization voltage, under which a stable and self-ordered growth of PAA can proceed, depends on the dissociation constant (pK_a_ = −log_10_K_a_) of a given acid [12,13]. Therefore, the cell size of the self-ordered porous alumina shows a tendency to increase with an increase in the pK_a_ value (i.e., lower acidic strength). On the other hand, the applied voltage determines the interpore distance (D_c_) in PAAs. In conventional anodization conditions, the D_c_ is linearly proportional to the anodizing potential (D_c_ = kU), with proportionality constants k ~ 2.5 nm/V for acidic electrolytes [14], or k ~ 3.0 nm/V for alkaline solutions [15]. Consequently, to obtain PAAs with a large cell size (D_c_ > 380 nm—an approximate lower limit of visible spectral range), anodization must by performed under high voltages using relatively weak acids. High-voltage anodization (associated with high electric field strength, E), in turn, is usually accompanied by intensive Joule heat evolution and large stress generation during the pore formation at the metal–oxide interface. As an effect, it is difficult to maintain the process stability. Moreover, for applications in photonics or optics, other effects frequently encountered during anodization at high E, such as plastic deformation of aluminum substrate or non-uniform PAA thickness [16,17], need to be avoided. 

Etidronic acid offers a possibility of obtaining highly regular PAA with large D_c_. Anodization in etidronic acid was first demonstrated by Kikuchi et al. [18,19,20]. Although its dissociation constant is quite high (pK_a1_ ~ 1.4 [21]), the application of a special anodization strategy has allowed for application of voltages in the 210–270 V range [18]. In order to enable a stable anodization at high potentials, the process was always started at a lower voltage, which was next linearly increased to the target values. As a result, the PAA with D_c_ in the range 530–670 nm was fabricated in a 0.3 M etidronic acid solution and various anodizing temperatures (20–60 °C). It was shown that the highest (critical) anodizing voltage for the steady-state growth of anodic porous alumina decreased as the electrolyte temperature increased. Furthermore, the electrolyte concentration was explored in the range of 0.2–4.2 M to establish the self-ordering conditions, including the range of anodization voltage and temperature, for a given acid concentration [19]. The cell size in this study ranged between 400 and 640 nm. Two-step anodization resulted in a perfect alignment of pores, with the diameter depending on the applied voltage, electrolyte concentration and post-synthesis pore-widening time. In order to arrange pores into the hcp structure extended over a large area (large domains), anodization has to be performed at a relatively high temperature using a relatively high concentration of etidronic acid. The process under these conditions is prone to instabilities, which can lead to dielectric breakdown and ultimately to sample burning [22,23]. To stabilize the process, an unique experimental setup with efficient heat dissipation needs to be employed.

Another way to prevent the burning phenomena is to use electrolyte modifiers, such as ethanol. Ethanol was used a cooling agent to prevent the catastrophic flow of current under high current anodization conditions and to enable the process to proceed at subzero temperatures [12,24,25]. Lower anodization temperature reduces the ion diffusivity. Therefore, relatively fewer acid anions can reach the barrier layer. It is also believed that the vaporization of ethanol takes away heat generated at the metal/oxide interface, thus keeping a lower temperature at the pore base [26,27]. Apart from the cooling effect, it was demonstrated that the addition of modifiers into an electrolyte can significantly change the process and influence the growth of PAA membranes [28]. The addition of ethanol was effective in suppressing the chemical dissolution of alumina into the electrolyte, thus improving the efficiency of the film formation (the ratio of the formed Al_2_O_3_ to the consumed Al) under a constant charge [29,30,31]. Thanks to the lower dissociation power of the modified electrolyte, the maximal attainable PAA thickness was increased. A similar trend was also observed for other additives, such as methanol, ethylene glycol, and glycerol, with the best efficiency of alumina formation and the lowest dissolution potential of electrolyte assessed for ethylene glycol [31]. For 50 vol% ethylene glycol added to sulfuric electrolyte, PAA with less than 10% porosity was formed. Furthermore, no influence of ethanol on PAA growth rate was found in these works [29,30]. In contrast to those results, Qin et al. [32] have shown a strong impact of ethanol on PAA growth rate. A dual effect of ethanol addition on PAA formation depending on the amount of ethanol added to the electrolyte was observed. The oxide growth rate increased five times when the amount of ethanol was about 10%. The opposite trend was recorded for higher ethanol concentration. Regardless of the discrepancies, the results demonstrate that ethanol not only stabilizes the temperature at the anode but can also substantially affect the efficiency of the porous alumina formation. The efficiency of PAA growth, in turn, translates into its porosity, which is a very important factor that determines further application of the template (e.g., refractive index of a porous layer): the higher the efficiency, the lower the porosity, and vice versa [33]. Moreover, it has been shown before that the addition of ethanol has an impact on the D_c_ and pore arrangement [34]. For the samples prepared in ethanol-modified electrolyte, PAA with slightly larger D_c_ and lower pore ordering was formed. Therefore, using ethanol as a stabilizer must be associated with detailed knowledge on other possible changes that can be induced by this modifier when introduced to the electrolyte. In particular, fully controllable electrochemical conditions to obtain PAA with precisely predicted cell size, porosity, and pore ordering are very desirable for designing 1D photonic crystals based on the PAA [35,36].

In this work, we systematically analyzed the influence of ethanol (EtOH) on PAA growth in etidronic acid solution. Three different electrolytes were studied: pure water-based etidronic solution (0:1 *v*/*v* EtOH:H_2_O), and ethanol-modified solutions with 1:9 and 1:3 *v*/*v* EtOH:H_2_O proportion. The second anodization was conducted at different temperatures in the range of 0–20 °C. It is shown that the porosity of PAA increases with temperature; however, the dissolution strength of the electrolyte is weakened in the 1:3 *v*/*v* EtOH:H_2_O solution. Moreover, the PAA thickness vs. charge-density dependence suggests that the alumina formation efficiency starts to be larger above 10 °C for the latter electrolyte as compared to the other two electrolytes. The addition of ethanol stabilizes the PAA formation process, which is visible in the lower currents evolving during the anodization. However, a quantitative analysis of pore arrangement demonstrates that ethanol slightly impacts hcp pore ordering. The results presented in this work may be very useful for designing PAA with a large cell size (D_c_ > 540 nm) and good pore regularity for optical or photonic applications.

## 2. Materials and Methods

**Figure 1:**
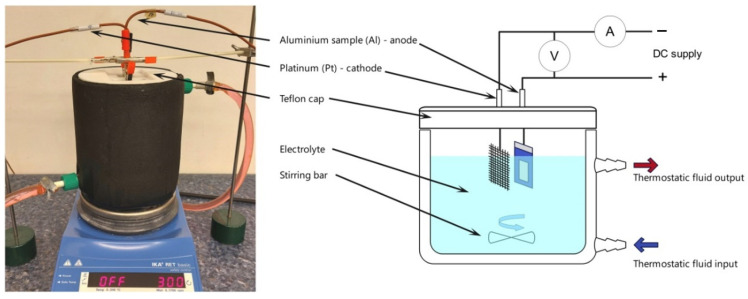
Experimental setup (on the left side—a photo of the electrochemical cell; on the right side—a scheme of the electrochemical units) used during anodization.

High-purity Al foil (99.9995% Al, Goodfellow, Huntingdon, UK) with a thickness of about 0.25 mm was cut into rectangular specimens (2 × 1 cm). Before the anodization process, the Al foils were degreased in acetone and ethanol and subsequently electropolished in a 1:4 mixture of 60% HClO_4_ and ethanol at 0 °C, under constant voltage of 25 V, for 2.5 min. Next, the samples were rinsed with a distilled water and ethanol and dried. As-prepared Al specimens (rectangular shape) were insulated at the back and the edges with acid-resistant tape, and served as the anode. A Pt grid (rectangular shape) was used as a cathode and the distance between both electrodes was kept constant (ca. 2 cm). Pt/Al electrode area ratio was about 25. Etidronic acid 60% aqueous solution was purchased from Merck KGaA (Darmstadt, Germany). A large 1L electrochemical cell and cooling bath thermostat (model MPC-K6, Huber company, Offenburg, Germany) were employed in the anodizing process. An adjustable DC power supply with voltage range of 0–300 V and current range of 0–5 A, purchased from NDN, model GEN750_1500 TDK Lambda, TDK Co., Tokyo, Japan, was used to control the applied voltage. RIGOL DM 3058E digital multimeter (Portland, OR, USA) was used to measure and transfer the registered current to a computer. A scheme of the experimental setup is shown in Figure 1.

In anodization, 0.3 M etidronic acid solution was used. In the first anodization step, the voltage (U) of 80 V was applied at first, which was maintained for 3 min and then stepwise raised to 210 V. Next, a constant anodization at 210 V was performed for 3 h. A temperature of 38 °C was used in the first step. After the process, alumina was chemically removed using a mixture of 6 wt% phosphoric acid and 1.8 wt% chromic acid at 60 °C for 120 min. In the second step of anodization, temperature was varied between 0 and 20 °C. Moreover, in order to keep the starting voltage close to the target one, anodization was started at U = 150 V, which was at once linearly increased to 210 V. After reaching 210 V, anodization was continued for 3 h. The duration of the process was always the same in order to eliminate the effect of oxidation time on the PAA thickness. Three etidronic acid solutions were studied: 0:1 *v*/*v* EtOH:H_2_O, 1:9 *v*/*v* EtOH:H_2_O, and 1:3 *v*/*v* EtOH:H_2_O.

Morphological analysis was performed using a field-emission scanning electron microscope FE-SEM (AMETEK, Inc., Montvale, NJ, USA). To obtain the interpore distance (D_c_) of the fabricated samples, Fast Fourier transforms (FFTs) were generated based on three SEM images taken at the same magnification for every sample, and were further used in calculations with WSxM software (version 5.0) [37]. The average D_c_ was estimated as an inverse of the FFT’s radial average abscissa from three FE-SEM images for each sample. Pore diameter (D_p_) was calculated with the use of NIS-Elements image analysis software (Nikon, Tokyo, Japan). Porosity (P) of the PAA membranes was calculated using the following formula: P = $\frac{\pi }{2\sqrt{3}}{\left(\frac{D_{p}}{D_{c}}\right)}^{2}$ [14]. The pore ordering was calculated using a home-made template made in OriginLab software (OriginLab 2022, OriginLab Corp., Northampton, MA, USA).

Conductivity of the electrolytes was measured in a thermostatic cell with Elmetron CC 505 conductivity meter, Zabrze, Poland. At the end, average values from three measurements were given.

## 3. Results and Discussion

**Figure 2:**
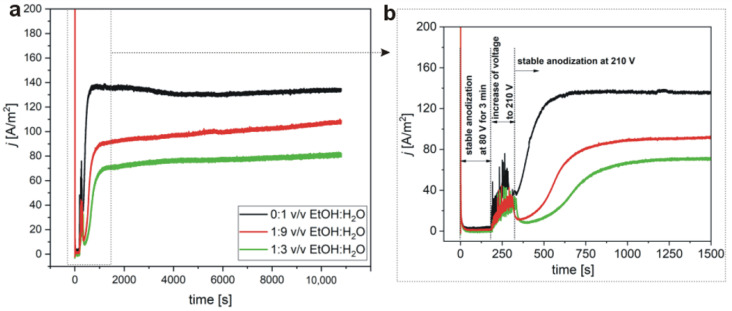
Current density vs. time curves recorded during I anodization at 38 °C in 0.3 M etidronic electrolytes without and with different amount of ethanol (**a**); a larger magnification of the j(t) curves (**b**).

Current density (j) vs. time (t) transients recorded during first anodization in 0.3 M etidronic acid solution, without and with different amounts of ethanol (EtOH), are shown in Figure 2. The process is divided into three stages (Figure 2b). In the first stage, a stable anodization at 80 V for 3 min was performed, where currents were stabilized at values close to zero after a sharp rise at the very beginning of the process. This behavior signalizes a water–electrolytic processes and the formation of the barrier layer afterwards [38,39]. In the second stage, voltage was raised to 210 V, accompanied by a turbulent j increase. The turbulences were caused by a temporary barrier layer thinning (stronger field-assisted oxide dissolution). It is also probable that a local dielectric breakdown occurred owing to the sudden disruption of the barrier layer continuity, which was, however, immediately rebuilt by an accelerated Al oxidation at the Al/oxide interface due to the increased ion migration. After reaching the target voltage (the third stage), currents first decreased and then started to rise again to maximal values depending on the composition of the electrolyte: with an increasing amount of ethanol, the maximal j decreased. Furthermore, increasing the amount of ethanol delayed the moment of the j increase. In the third stage, the anodization was conducted under a stable target voltage (210 V), followed by the occurrence of a plateau in the j(t) curves. The current decrease in the third stage signalizes a thickening of the barrier layer to the value characteristic for the applied voltage (the thickness of the barrier layer is proportional to the applied voltage [9]), whereas the subsequent current rise indicates the commencement of pore nucleation. The plateaus in the j curves reflect the stabilization between the oxide growth at the aluminum/oxide interface and the oxide dissolution at the oxide/electrolyte interface [39].

**Figure 3:**
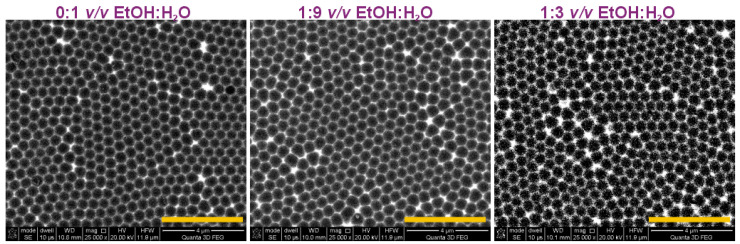
SEM images of Al substrates after dissolution of anodic alumina formed in I anodization, performed in 0.3 M etidronic acid solutions without (0:1 *v*/*v* EtOH:H_2_O) and with two different amount of ethanol: 1:9 *v*/*v* EtOH:H_2_O and 1:3 *v*/*v* EtOH:H_2_O (the yellow scale bar = 4 µm).

In Figure 3, SEM images of the Al substrates after dissolution of the alumina produced in the first anodization step are demonstrated. Highly regular, hexagonal-shape concave arrays were formed in the sample produced in the pure water-based etidronic electrolyte (0:1 *v*/*v* EtOH:H_2_O). The addition of ethanol makes the arrangement of dimples slightly less ordered. In the sample produced in the 1:3 *v*/*v* EtOH:H_2_O solution, the domains with close-packed hexagonal pores were visibly smaller than those formed in the electrolyte without ethanol. At the same time, larger areas of pores with an irregular shape (pentagons, heptagons, etc.) that are not surrounded by exactly six near-neighbors were observed. The deterioration of pore arrangement upon addition of the additive was also observed during hard anodization in an oxalic electrolyte [34]. The effect may come from the lower currents, and thus lower electric field strength across the barrier layer, generated during anodization in electrolytes modified by the additive. The lower electric field strength, in turn, may contribute to smaller compressive stresses exerted on neighboring pores. As a result, the pores tend to lose their hexagonal arrangement [40].

**Figure 4:**
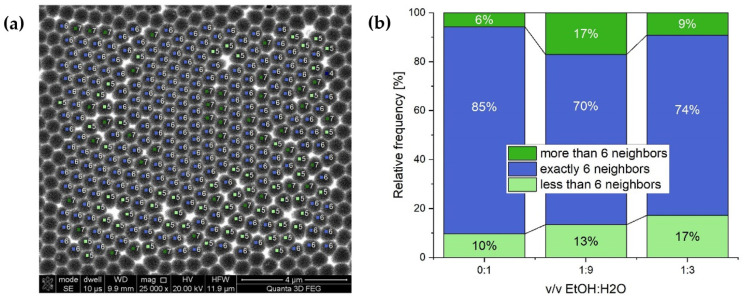
Presentation of the pore-ordering calculation method: blue—a pore with exactly six neighbors; light green—a pore with less than six neighbors; dark green—a pore with more than six neighbors (**a**); and the ordering degree pores after I anodization expressed as a relative frequency of the pores with 6, <6, and >6 nearest neighbors (**b**).

The ordering quality of porous patterns in anodic oxide samples prepared in different solutions was also quantitatively determined by a method adapted from Hillebrand et al. [41]. In the article [41], a cut-off radius is determined around each pore, which determines the closest neighbors. In this work, the cut-off radius was used to calculate the number of nearest neighbors (Figure 4a). The number of the closest neighbors determined the ordering degree of the porous structure expressed in relative frequency (the number of pores in [%]) of pores with exactly 6, <6, and >6 nearest neighbors. Therefore, the highest [%] of the pores with six neighbors indicates the highest pore ordering level. Figure 4b shows the relative frequency of pores with different numbers of nearest neighbors. It can be seen that the sample produced in the electrolyte without ethanol (0:1 *v*/*v* EtOH:H_2_O) possesses the highest level of hexagonal pore ordering (85% of relative frequency of pores with 6 nearest neighbors).

**Figure 5:**
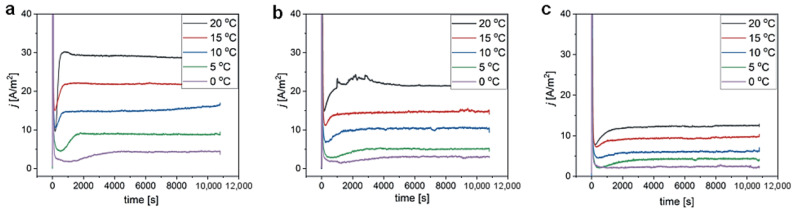
Current density (j) vs. time curves recorded during II anodization at 210 V at various temperatures in 0.3 M etidronic acid solutions without ethanol (**a**), and in the 1:9 *v*/*v* EtOH:H_2_O (**b**) and 1:3 *v*/*v* EtOH:H_2_O (**c**) mixtures.

The j(t) transients recorded during II anodization at temperature (T) ranging between 0 and 20 °C are gathered in Figure 5. At the beginning of the process, a sharp rise of j is observed, which can be ascribed to the occurrence of a water–electrolytic reaction. Immediately afterwards, a barrier layer starts to form, which is shown through a sharp drop of the j. Pore nucleation and growth are shown by a rise of current until steady-state conditions (a balance between formation and dissolving of alumina) are reached (a plateau in the j(t) curves). The current density decreases with the addition of ethanol and with lowering anodizing temperature. Both effects can be associated with lower chemical dissolution power of the electrolyte that makes the barrier layer thicker, thus reducing the migration of ions (e.g., Al^3+^, O^2−^) and thus the steady currents [12,27]. Moreover, ethanol is a cooling agent that generates lower Joule heat at the oxide/metal interface due to its high effumability [24,25]. Lower temperature in the reaction center, in turn, contributes to the lower ion migration rates (lower currents). 

Additionally, because of the lower dielectric constant of ethanol compared to that of water, the dielectric constant of an ethanol–water mixture decreases with the addition of ethanol [42]. The lower dielectric constant of the electrolyte suppresses the dissociation of the acid in the mixed solution, resulting in a lower concentration of acid anions at the barrier layer and consequently in the lower currents [8]. It can be also observed that the stages of pore nucleation and subsequent process stabilization are delayed by the increasing amount of ethanol added to the electrolyte, as well as by the decreasing anodizing temperature. In the curve recorded during anodization in the 1:3 *v*/*v* EtOH:H_2_O solution at 0 °C, the rise of current after the barrier layer formation is hardly visible, indicating early stages of pore nucleation and development. 

**Figure 6:**
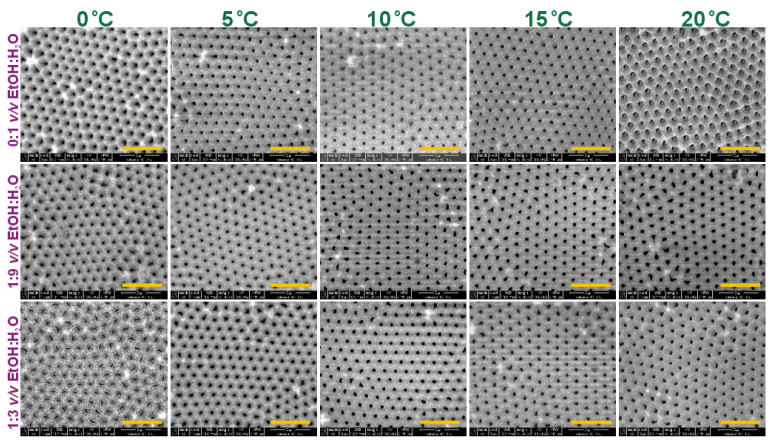
SEM images of porous anodic alumina formed during II anodization performed in 0.3 M etidronic acid solutions without (0:1 *v*/*v* EtOH:H_2_O) and with two different amounts of ethanol: 1:9 *v*/*v* EtOH:H_2_O and 1:3 *v*/*v* EtOH:H_2_O, and in the anodizing temperature between 0–20 °C (the yellow scale bar = 2 µm).

**Figure 7:**
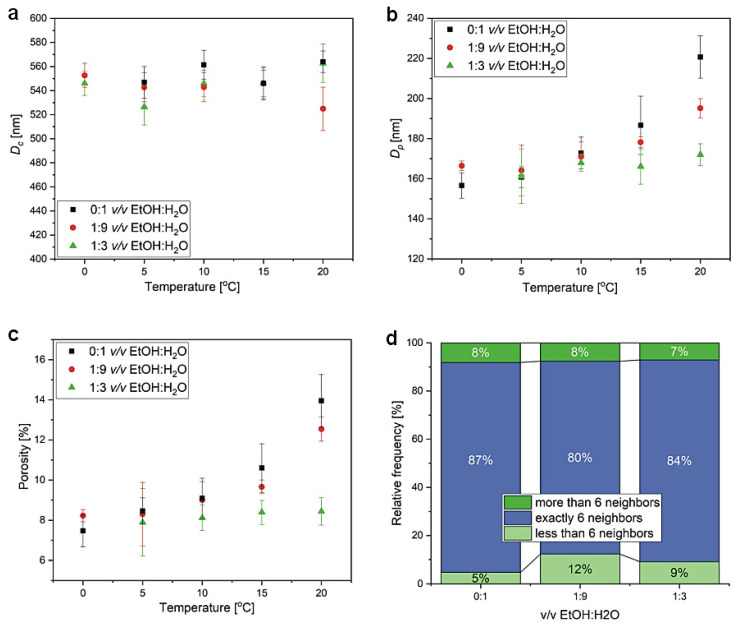
Interpore distance (D_c_) (**a**), pore diameter (D_p_) (**b**), porosity of PAA (**c**) vs. temperature for different ethanol fractions; and the ordering degree of pores after II anodization calculated for PAAs formed at 15 °C (**d**).

In Figure 6, SEM images of the porous alumina formed in the second anodization step are shown. The sample prepared in 1:3 *v*/*v* EtOH:H_2_O at 0 °C is characterized by narrow slit-like pores, which evidences the beginning of the pore development process and corresponds well with the j(t) behavior recorded for this PAA (Figure 5c). Based on the images, interpore distance (D_c_), pore diameter (D_p_), and porosity of the PAA templates (except for the PAA produced in 1:3 *v*/*v* EtOH:H_2_O at 0 °C) were determined (Figure 7a–c). The D_p_(T) dependence for PAA obtained in a pure water-based electrolyte was also studied in 0.3 M oxalic electrolyte [27]. In the oxalic solution, the pore diameter increased over two times when anodizing temperature was increased from 0 to 20 °C, whereas the change of D_p_ in the etidronic electrolyte was more gentle (from around 150 to 220 nm in the same temperature range). The slightly less-pronounced effect of temperature on pore diameter in the etidronic electrolyte may be due to a slightly higher dissociation constant of etidronic acid (pK_a1_ = 1.35) compared to that of oxalic acid (pK_a1_ = 1.25) [21]. Porosity increases with anodizing temperature for the PAA anodized in etidronic electrolyte without ethanol and in the 1:9 *v*/*v* EtOH:H_2_O composition. However, the temperature effect is not so visible in the PAA produced in the 1:3 *v*/*v* EtOH:H_2_O mixture. It can be observed that at a temperature higher than 10 °C, ethanol demonstrates a suppression effect for the chemical dissolution of the oxide layer, while in the PAAs produced in the 1:9 *v*/*v* EtOH:H_2_O and 0:1 *v*/*v* EtOH:H_2_O solutions, porosity starts to sharply rise above 10 °C, and in the PAAs formed in 1:3 *v*/*v* EtOH:H_2_O solution the porosity remains more or less the same. Smaller porosity is directly related to a larger portion of Al^3+^ cations that is used to form Al_2_O_3_ instead of being rejected to electrolyte [33]. Therefore, the smaller porosity gives an indication about the higher oxide formation efficiency in the electrolyte with the largest amount of ethanol.

Furthermore, the difference in pore regularity induced by ethanol seems to be weakened after the II anodization: the level of pore ordering looks comparable regardless of temperature applied (Figure 6). Pore-ordering level was determined for the samples anodized at 15 °C. It can be seen that the level was still the best for the PAA produced in a pure water-based solution (Figure 7d). Moreover, the relative frequency of pores with exactly six neighbors after the II anodization was higher than that after the I anodization (Figure 4b) in all studied samples. However, in the sample fabricated in the electrolyte without ethanol (0:1 *v*/*v* EtOH:H2O), the increase was insignificant (approx. 2%), whereas in the samples produced in the electrolytes with ethanol the increase was much greater (approx. 10%).

**Figure 8:**
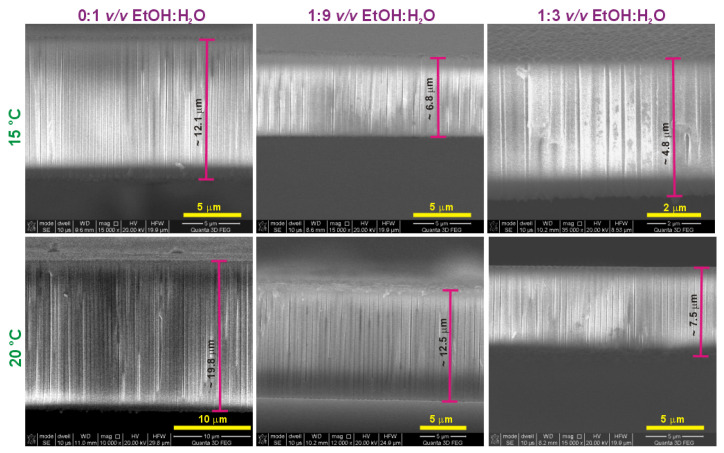
Cross-sectional views of PAAs produced in 0.3 M etidronic acid solutions without (0:1 *v*/*v* EtOH:H_2_O) and with two different amounts of ethanol: 1:9 *v*/*v* EtOH:H_2_O and 1:3 *v*/*v* EtOH:H_2_O at temperature of 15 (first row) and 20 °C (second row).

**Figure 9:**
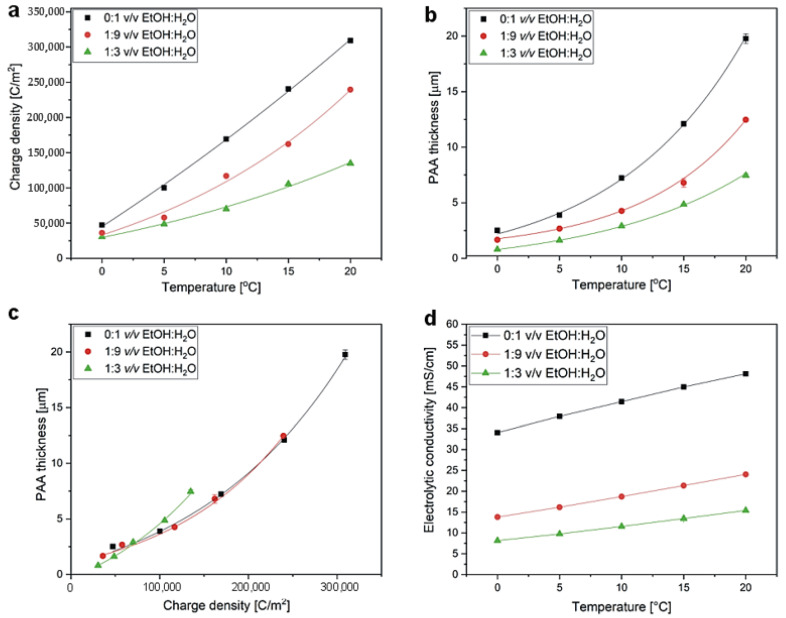
Charge density (**a**) and PAA thickness (**b**) vs. anodizing temperature; PAA thickness vs. charge density (**c**); and electrolytic conductivity of 0.3 M etidronic electrolytes of different composition vs. temperature (**d**).

In order to further analyze the efficiency of PAA formation in the etidronic solutions of different compositions, charge density, as well as the thickness of PAA formed in each process, were determined. In Figure 8, cross-sectional views of selected PAA templates are gathered, showing perfectly aligned and parallel pores throughout the whole membrane thickness. Charge density and the thickness of PAA as a function of anodizing temperature are shown in Figure 9a,b, respectively. Since PAA thickness increases with the charge passing during the anodization with the proportionality coefficient that depends on the electrochemical conditions [43], both charge density and PAA thickness exhibit an exponential relation with anodizing temperature according to the Arrhenius equation (the points were plotted with exponential functions). This is because the temperature activates the migration of ions though the barrier layer, and thus increases the charge flow and the PAA growth rate. The same oxide growth efficiency implies that the same oxide thickness is attained for the same charge density value, regardless of the electrochemical conditions, including the type of electrolyte [43]. In Figure 9c, the PAA thickness is juxtaposed with the charge density determined for the processes conducted at increasing temperature. It can be observed that for the samples grown in the 0:1 *v*/*v* EtOH:H_2_O and 1:9 *v*/*v* EtOH:H_2_O etidronic electrolytes, the oxide thickness is exactly the same for the whole charge density range, whereas in the 1:3 *v*/*v* EtOH:H_2_O solution the PAA thickness increases above a certain charge density that corresponds to anodizing temperature of 10 °C. This result, together with the observed smaller porosity in the PAAs produced in the 1:3 *v*/*v* EtOH:H_2_O solution (Figure 7c), strongly suggests that the PAA formation efficiency increases at T > 10 °C in the etidronic electrolyte with the largest amount of ethanol. The higher efficiency of the oxide formation upon the addition of the alcohol was also found in the oxalic and sulfuric electrolytes [29,30,31]. As indicated in previous works (e.g., [12,27]), ethanol is a cooling agent that evaporates at the bottom of pores due to the high effumability taking away the heat generated at the reaction spot and preventing the increase of the localized temperature. These processes suppress the chemical dissolution of the oxide layer. The weaker chemical dissolution means that more Al^3+^ cations are retained in the alumina instead of being rejected to the electrolyte, leading to the thicker PAA for the same charge density (higher oxide formation efficiency). In other words, upon addition of a certain amount of ethanol (1:3 *v*/*v* EtOH:H_2_O or more), the oxide growth at the aluminum/oxide interface occurs faster than the oxide dissolution at the oxide/electrolyte interface.

The current density is strictly related to the ion mobility in a solution. The conductivity (σ) of the electrolytes measured in different temperature is shown in Figure 9d. The σ corresponds well with the current density behavior: both increase with temperature for all studied solutions. The currents that are recorded during the anodization of aluminum are composed of ionic (outward Al^3+^ motion and inward motion of various anions: O^2−^, OH^−^, electrolyte-anion contaminant species) and electronic parts, resulting from complex reactions to form anodic oxide driven by the external electric field [44]. These reactions are accelerated when the temperature of the electrolyte rises. Consequently, the higher reaction rates increase the motion of the ions, the PAA growth rate and the current densities. It must also be noted that, as a cooling agent, ethanol neutralizes the effect of the increasing temperature. As an effect, both σ and currents do not change so much with the temperature in the electrolytes with the modifier compared to the electrolyte without ethanol. The behavior may be related to the hydrodynamic radius of the etidronic ion with coordination spheres, including solvent particles attached to the ion (water and ethanol). Ethanol can additionally enlarge the sphere, reducing the ionic mobility of the etidronic ions [45] and thus contributing to the observed behavior. Last but not least, the increase of σ and j(t) may be also associated with the viscosity of a solvent. The viscosity decreases strongly when temperature is increased [46], further improving the ionic mobility. Similarly, the decreases of σ with the addition of ethanol can be due to the increase of the viscosity of the solution with an increasing amount of ethanol [47]. Moreover, with increasing concentrations of ethanol, the dielectric constant of the solvent decreases [48,49]. This decrease in the dielectric constant makes it more difficult for the dissociation reaction of an acid to proceed in a medium containing ethanol. Owing to the reduction of the concentration of dissociated ions, the conductivity of the solution decreases. Additionally, this suppression of the dissociation of etidronic acid with ethanol is probably the primary reason for the current density and conductivity performance (both decrease with the addition of an increasing amount of ethanol).

## 4. Conclusions

The production of porous anodic alumina (PAA) with large cell sizes that correspond to visible or infrared wavelengths remains a challenge to date because of the necessity of using high voltages, which make the process prone to burning and breakdown phenomena. Additionally, for applications in photonics or optics, PAAs should be characterized by high pore regularity. Self-ordered PAAs with a large cell size (>540 nm) were fabricated in etidronic acid solutions. To stabilize the process, different amounts of ethanol were added to the electrolyte. It is known that the additive can change the mechanism of pore formation and modify the PAA growth. Therefore, for the first time, the impact of ethanol on pore geometry and level of pore ordering was systematically analyzed in this study. It is shown using a quantitative method that the additive tends to lower pore ordering in PAA. The effect might be linked with lower electric field strength (lower current densities) and, consequently, lower stresses exerted on the neighboring pore, which, as a result, start to lose their ordering power. The second anodization was conducted at various temperatures ranging from 0 to 20 °C. The PAA growth rate increases exponentially with temperature and the PAA thickness is proportional to the charge passing during the process. It was, however, revealed that proportionality between PAA thickness and charge density was slightly different for the samples produced in the electrolyte with the largest amount of ethanol (1:3 *v*/*v* EtOH:H_2_O). This observation, together with the lower porosity determined for the corresponding PAA samples, strongly suggests that the PAA formation efficiency increased in the 1:3 *v*/*v* EtOH:H_2_O electrolyte above 10 °C. The higher alumina growth efficiency could be related to a suppression effect for the chemical dissolution of the oxide layer demonstrated by ethanol. The results show that depending on the amount of ethanol added to the electrolyte and anodizing temperature, the porosity of PAAs can be adjusted within a certain range of variability. At 20 °C, porosity dropped from about 14% in the pure water-based etidronic electrolyte to ca. 8% in the 1:3 *v*/*v* EtOH:H_2_O etidronic solution. Future studies will involve optimizing the process at higher voltages (even up to 300 V) using ethanol or other modifiers in order to increase the cell size while maintaining a high level of pore ordering. The work can be very helpful in designing photonic materials based on PAAs with large cell sizes (D_c_ > 540 nm). 

## Data Availability

Not applicable.
